# A Comparison of Intravenous plus Intraperitoneal Chemotherapy with Intravenous Chemotherapy Alone for the Treatment of Gastric Cancer: A Meta-Analysis

**DOI:** 10.1038/srep12538

**Published:** 2015-07-29

**Authors:** Sheng Yang, Rui Feng, Zhang-Chi Pan, Tao Jiang, Qian Xu, Qiang Chen

**Affiliations:** 1 Department of Medical Oncology, Fujian Medical University Union Hospital, Fujian, China; 2 Teaching and Research Department of Oncology, Union Clinical medical College of Fujian Medical University, Fujian, China; 3 Fujian Key Laboratory of Translational Cancer Medicine, Fujian, China; 4 Fujian Medical University Stem Cell Research Institute, Fujian, China

## Abstract

We aimed to evaluate the effectiveness and safety of intravenous (IV) plus intraperitoneal (IP) chemotherapy compared to intravenous (IV) chemotherapy alone for patients with gastric cancer. Electronic databases were searched up to June 2013. Two authors independently selected studies, extracted data and assessed the quality of included studies. The GRADE System was adopted to rate the level of evidence. Of 392 citations, five RCTs involving 1072 patients were included. Overall, a significant improvement in in one- and three- and five-year survival rate was observed in the IV plus IP chemotherapy group (3 RCTs, n = 360, RR = 1.10, 95% CI 1.04 to1.17), (5 RCTs, n = 953, RR = 1.22, 95% CI 1.11 to1.35) and (3 RCTs, n = 347, RR = 1.42, 95% CI 1.12 to 1.80), respectively. Results supported a significant decrease in the rate of metastases (1 RCT, n = 85, RR = 0.41 95% CI 0.19 to 0.89) and peritoneal recurrence (2 RCTs, n = 297, RR = 0.41, 95% CI 0.26 to 0.62) in the IV plus IP chemotherapy group, however, the incidence of adverse events was increased. For patients with gastric cancer, IV plus IP chemotherapy can improve the overall survival rate and prevent the distant or peritoneal metastases. An increased risk of neutropenia, peripheral edema and neuropathy was observed.

Gastric cancer is one of the most prevalent cancers worldwide and the second leading cause of cancer death^1^,^2^. In 2008, new cases and deaths resulting from gastric cancer were estimated to be 990,000 and 738,000, respectively^2^. At present, surgery remains the major therapeutic approach for resectable gastric cancers. However, 60% of patients who undergo resection will suffer from local relapse or distant metastases^3^, which negatively impact the survival time and quality of life.

For patients with advanced gastric cancer and metastases, standard surgery plus IV chemotherapy is an option. Based on the results of a previous meta-analysis that compared IV chemotherapy plus surgery versus surgery alone for patients with gastric cancer, surgery plus IV chemotherapy was found to potentially benefit patients. However, local recurrence and peritoneal metastases remained a challenge. IP chemotherapy was developed because of the difficulty in achieving therapeutic levels of chemotherapeutic agents within the abdominal cavity with IV administration. In recent years, certain RCTs have compared IV plus IP chemotherapy versus IV chemotherapy alone after resection. Diverging opinions have emerged within the medical community regarding the effectiveness and safety of these two therapeutic regimens. Several systematic reviews have evaluated the effect of IP chemotherapy as an adjuvant to surgery^4^,^5^,^6^,^7^,^8^,^9^. However, whether IP therapy should be added to IV chemotherapy after surgery has not been well explored. To determine if this combined chemotherapy is better than IV chemotherapy alone after curative resection, we collected the current evidence and included all relevant RCTs in this meta-analysis.

## Methods

This article was performed based on the guidance of the Preferred Reporting Items for Systematic Reviews and Meta-analysis (PRISMA) statement^10^ and the Cochrane Handbook for Systematic Reviews of Interventions^11^. All pooled analyses were based on published studies, and thus did not require ethical approval and patient consents.

### Literature search

Seven electronic databases were searched for studies published up until June 2013, including Medline, Embase, Web of Science, CENTRAL, ClinicalTrials.gov, Current Controlled Trials and CINAHL. The following search terms were used: operation, surgery, resection, infusion*, intravenous, intraperitoneal, chemotherapy, carcin*, cancer*, neoplas*, tumour*, cyst*, adenocarcin*, intestin*, digest*, grastr*, epigastr*, gut, stomach*. The reference lists of selected studies and relevant reviews were also manually searched to include any relevant articles, and the process above was performed repeatedly until no additional studies were identified. Conference abstracts meeting the inclusion criteria were also included.

### Selection Criteria

According to the PICOS acronym (Population, intervention, comparator, outcomes and study design), we defined the inclusion criteria as below: (1) Population (P): all patients that were diagnosed with gastric cancer based on pathology, cytology and who were undergoing radical gastric resection (2) Intervention (I) and comparator (C): comparing the efficacy and safety of IV plus IP chemotherapy versus IV chemotherapy alone. (3) Outcomes (O): the following measured outcomes were included: overall survival, rate of metastases, recurrence, peritoneal recurrence, and adverse events. (4) Study design (S): RCTs.

The following references were excluded: (1) impossible to extract the data due to insufficient information and not able to acquire primary data from authors; (2) for different papers from the same study, the article with the strictest methodology and most complete data was chosen for incorporation.

### Data Extraction and Assessing the Risk of Bias

Two authors extracted the following information independently by using a predesigned form: first author, publication year, country, sample size, baseline characteristics of patients, diagnosis, length of illness, study setting and management of interventions. Continuous or binary data reported on specific outcomes was also extracted from the original studies. For data from multiple treatment groups, the approach recommended in the Cochrane handbook was adopted^11^ to avoid a unit- of -error analysis that may result from entering several comparisons into one meta-analysis, which could lead to ‘double-counts’ of patients based on the same study. When necessary, authors were contacted to acquire the complete data. Any divergence or disagreements between authors was resolved by consulting a third author.

Assessment of the risk of bias of the eligible studies was conducted by each author according to the Cochrane Handbook for Systematic Reviews of Interventions (Chapter 8)^12^. The evaluation index included randomization sequence generation, allocation concealment, blinding of participants and personnel, blinding of outcome assessors, incomplete outcome data, selective reporting and other biases. Based on the information extracted from primary studies, each domain was rated as “high risk”, “unclear risk” or “low risk”.

### Quality of the Evidence

The GRADE system is an approach to rating the level of evidence and the strength of recommendations, which is based on the risk of bias, imprecision, indirectness, inconsistency across studies, and publication bias. Two authors independently graded the evidence using GRADEpro software, version 3.6 [http://www.gradeworkinggroup.org/toolbox/index.htm]. Any disagreement was resolved by discussion with a third author.

### Statistical Analysis

All extracted data were entered into RevMan 5.2 (Copenhagen: The Nordic Cochrane Centre, The Cochrane Collaboration, 2012) for statistical analysis. For binary data, we calculated the relative risk (RR) and its 95% CI (confidence interval). Weighted mean differences (WMDs) or standard mean differences (SMD) with 95% CI for continuous outcomes were planned to select to estimate the pooled effects size; however, we did not encounter this type of data. Heterogeneity was evaluated based on the magnitude of the Chi^2^, corresponding P value and I^2^ statistic. Where the I^2^ was ≥50%, a random effects model based on Mantel-Haenszel (MH) or inverse variance (IV) statistical approach was selected to combine the data. If I^2^ was <50%, a fixed effects model based on MH or IV statistical approach was selected. Subset analysis was conducted based on the different measured time points of survival rate and type of metastases. We presented data on a “once-randomised-always-analye” basis. Those leaving the study early were all assumed to have the same rates of negative outcome as those who completed, by which, sensitivity analysis was conducted to detect the robustness of the result.

## Results

### Study selection and trial characteristics

**Figure 1:**
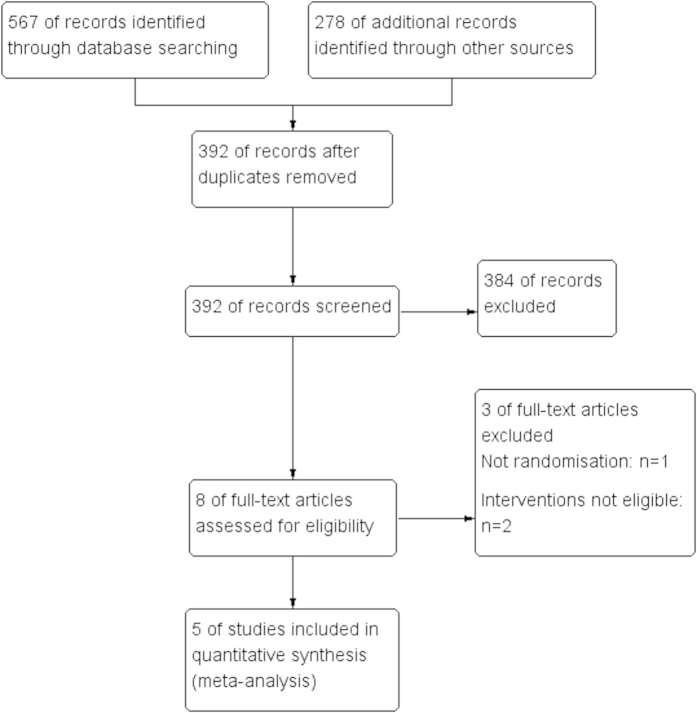
Flow Chart of Literature Retrieval and Trial Selection.

A total of 392 studies were identified from the initial literature search and five RCTs that included 1072 patients remained eligible for inclusion after screening. One study^13^ did not report the chemotherapeutic agents. The following agents were used in IV and IP chemotherapy: 5-FU, cisplatin, mitomycin-C, adriamycin and leucovorin. A flow diagram depicting the literature retrieval and trial selection is presented in Fig. 1. The characteristics of the five trials are summarized in Table 1.

### Assessment of risk of bias

**Figure 2:**
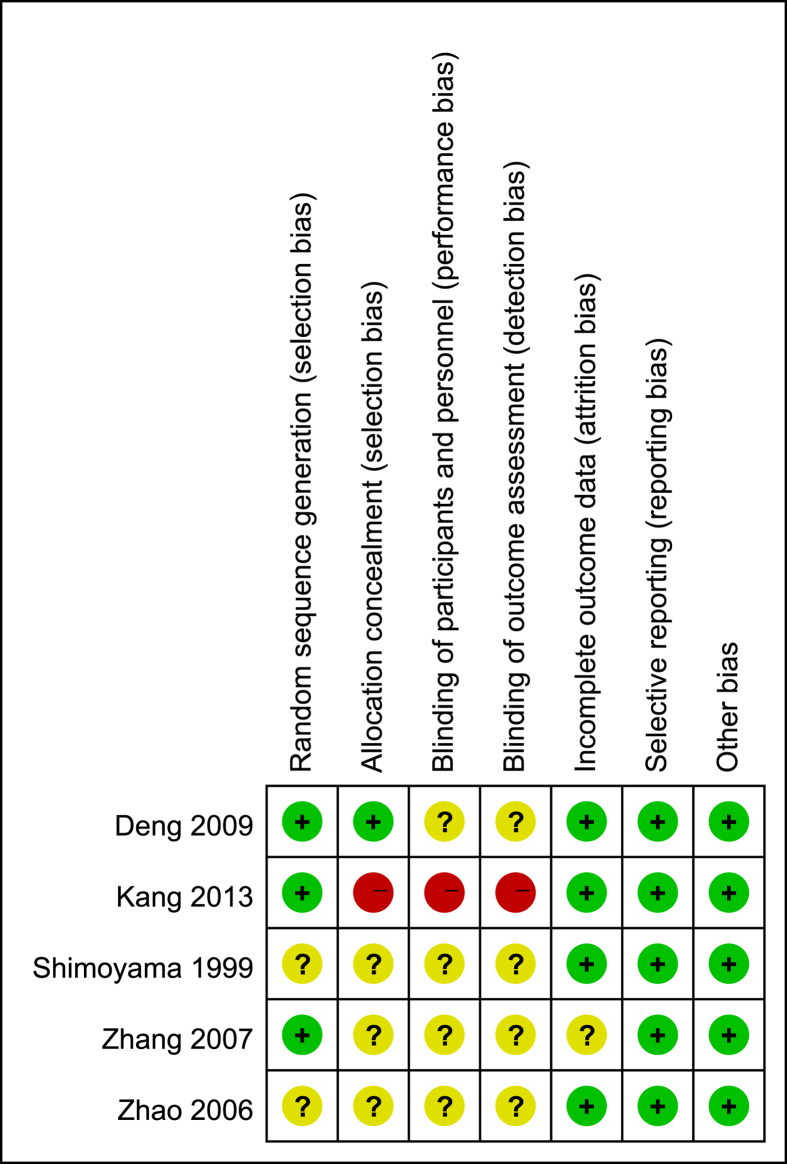
Risk of bias of individual study.

For the five included studies, three^14^,^15^,^16^ studies were rated as low risk in “randomization sequence”. The randomization was generated by using a random number table^16^ or permutation block randomization^15^. Patients were randomly assigned to accepted interventions in two studies. For the other two studies^13^,^17^, the author mentioned randomization, but the methods used to randomize study participants were unclear. Two^14^,^16^ studies were rated as low and high risk in allocation concealment respectively, mainly because the former used a sealed envelope while the latter was an open-label study. Four studies^14^,^15^,^16^,^17^ were rated as unclear risk in “blinding of participants and outcome assessor” and one was rated as high risk because of the open label design. Four studies^13^,^14^,^17^ were rated as low risk in “incomplete outcome data” and one^16^ was rated as unclear. All studies presented a complete list of patient data. One study^15^ was with less than 20% loss to follow-up (18%, 59/322 in the combined chemotherapy group and 18%, 60/318 in the IV alone group), which was rated as low risk of bias because the missing data was balance between group. No other potential bias was detected such as conflict of interest or early stopping of trials. The risk of bias of the included studies is summarized in Fig. 2.

### Survival rate

**Figure 3:**
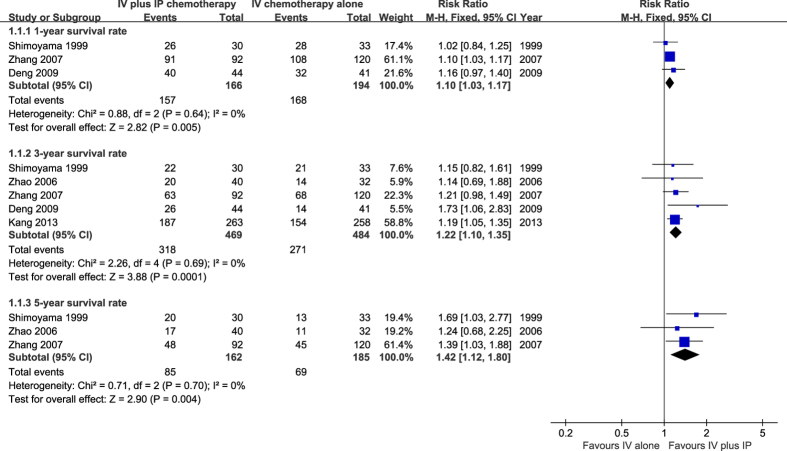
Meta-analysis on survival rate.

Three studies^13^,^14^,^16^ that included 360 patients reported the one-year survival rate. The meta-analysis revealed that there were significant differences between the two groups (RR = 1.10, 95% CI 1.03 to 1.17, P = 0.005) with the result in favor of the treatment group (IP plus IV chemotherapy). No heterogeneity was detected between studies (χ^2^ = 0.88, P = 0.64, I^2^ = 0%), therefore, a fixed effect model was selected (Fig. 3).

The three-year survival rate was reported in five studies^13^,^14^,^15^,^16^,^17^ that included 953 patients. There was no heterogeneity between studies (χ^2^ = 2.26, P = 0.69, I^2^ = 0%), and the fixed effect model was adopted to pool the results. The overall estimates suggested that the 3-year survival rate in the IV plus IP chemotherapy group was superior to that in the IV chemotherapy alone group (RR = 1.22, 95% CI 1.10 to 1.35, P = 0.001) (Fig. 3).

The data on the five-year survival rate can be extracted from three trials^13^,^16^,^17^ (347 patients). There was no significant heterogeneity identified (χ^2^ = 0.71, P = 0.70, I^2^ = 0%), and thus the fixed effect model was used. The result showed that the 5-year survival rate in the combination therapy group was higher than that in the IV chemotherapy alone group (RR = 1.42, 95% CI1.12 to 1.80, P = 0.004) (Fig. 3).

### Sensitivity analysis

**Figure 4:**
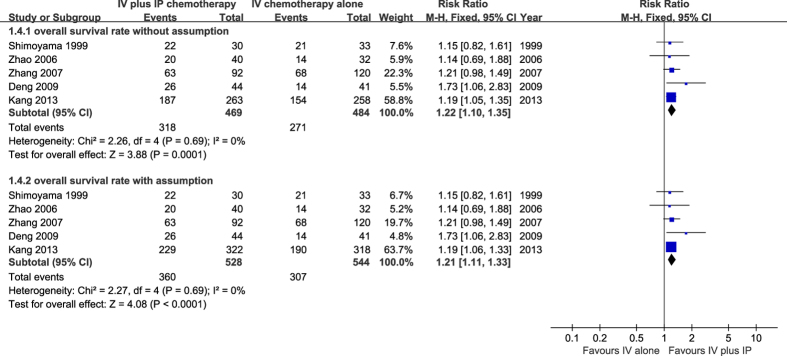
Sensitivity analysis survival analyses. We dealt the missing data with intention to treat analysis to test the robustness of result: we assumed that the patients who did not complete the study experienced the same rate of events with completers. The combined result with assumptions (Analysis 1.4.2) was consistent with result based on completers (Analysis 1.4.1).

For Kang, Y. K., *et al.*^15^ with 18% of drop-outs in each group, the sensitivity analysis was applied to test the robustness of the pooled overall survival rate. The missing data was assumed to be with the same overall survival rates as those who completed [71.1% (187.263) in the combined chemotherapy group and 59.7% (154/258) in IV alone group]. There was no significant heterogeneity across studies, so the fixed effect model was used. The results showed that there was no difference in the estimate of effects as a result of including or excluding the assumed data, suggesting that incomplete data did not elicit further bias (Fig. 4).

### Rate of metastases

**Figure 5:**
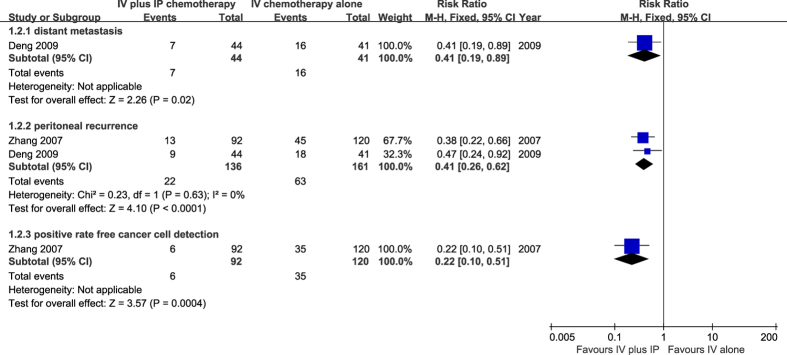
Meta-analysis on metastases rate.

One study^14^ involving 85 patients reported a rate of distant metastases within 2 years after surgery. Seven patients in the combination therapy group and 16 patients in the IV chemotherapy alone group developed distant metastases.. The result presented a significant lower risk of distant metastases in the combination therapy group (RR = 0.41, 95% CI 0.19 to 0.89, P = 0.002). Two studies^14^,^16^ that included 297 patients reported the incidence of peritoneal recurrence. The numbers of peritoneal recurrence were 22 and 63 patients in the combination therapy group and IV chemotherapy alone group, respectively. There was no heterogeneity between studies (χ^2^ = 0.23, P = 0.63, I^2^ = 0%), and a fixed effect model was selected. The results demonstrated that the combination therapy effectively decreased the peritoneal recurrence rate (RR = 0.41, 95% CI 0.26 to 0.62, P < 0.00001). In one study^15^ involving 212 patients that compared the detection rate of cancer free cells in peritoneal lavage, the overall estimate showed that patients in the treatment group had lower risk of this outcome (RR = 0.22, 95% CI 0.10 to 0.51, P = 0.004) (Fig. 5).

### Adverse reactions

**Figure 6:**
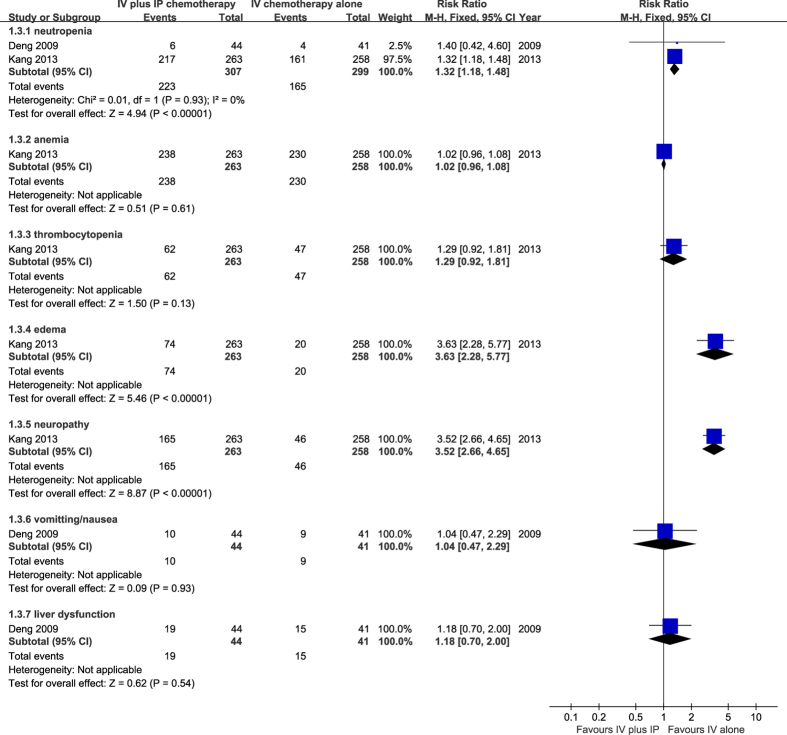
Meta-analysis on adverse events.

The incidence of adverse events such as marrow suppression (neutropenia or anemia), thrombocytopenia, peripheral edema, neuropathy, gastrointestinal reaction, and liver dysfunction were evaluated during the treatment period. Two studies^14^,^15^ reported adverse reactions resulting from the chemotherapy regimens. The result suggested a significantly greater risk of developing neutropenia in the combination therapy group (RR = 1.32, 95% CI 1.18 to 1.48, P = 0.001). The incidence of anemia and thrombocytopenia was not significantly different between both groups^9^. The rate of peripheral edema was also examined by Yoon-Koo, and the results showed a significantly increased risk of developing peripheral edema when receiving IV combined with IP chemotherapy (RR = 3.63, 95% CI 2.28 to 5.77, P = 0.001). The incidence of neuropathy in the treatment group was also greater than that in the IV chemotherapy alone group (RR = 3.52, 95% CI 2.66 to 4.65, P = 0.001). The incidence of nausea, vomiting and liver dysfunction was similar between the groups (Fig. 6).

### Level of evidence

Three outcomes were reported in this article. The survival rate was considered critical, and the rate of metastases and adverse reactions were also important findings. The level of the evidence of each result was presented in Table 2.

## Discussion

IV chemotherapy alone has limited effects on peritoneal recurrence and metastases because of the absence of vasculature in peritoneal metastatic tumor nodules^18^. IP chemotherapy has been adopted to treat several intra-abdominal cancers^19^,^20^,^21^ because of the greater ability to obtain an effective and prolonged drug concentration in the peritoneum than what can be achieved with IV chemotherapy. The peritoneal barrier consisting of mesothelium and underlying sub-mesothelium also slows the drug’s clearance^22^. This allows the chemotherapeutic agents to surround and “bathe” a tumor burden within the cavity. The medication is then absorbed into local tissue, including the primary tumor, directly affecting the tissue and cells it passes through^23^. Moreover, the agents absorbed from the peritoneal cavity also result in systemic absorption and create a blood level of the chemotherapeutic agent^24^, thereby synergistically enhancing the antitumor effect of IV chemotherapy.

Our meta-analysis showed that IV plus IP chemotherapy is a scientific and effective therapeutic approach that improves survival rate, especially at 3- and 5-years (67.8% vs. 56% and 52.5% vs. 37.3%, respectively). The 1- year survival rate in the treatment group is also slightly higher (94.6% vs. 86.6%). Individuals who received IV plus IP chemotherapy also had a lower relapse rate within three years after resection.

The rate of distant metastases was found to be significantly higher in the control group without IP treatment (39% vs. 15.9%). However, this conclusion should be viewed with caution, primarily because of the relatively small sample size (n = 85) and the potential selection and detection bias. Local recurrence is the main reason reported for treatment failure. As early as 1982, studies confirmed that the detection of partial residual or recurrence by autopsy was near 80% in gastric cancer patients. Our meta-analysis showed a lower incidence of local recurrence in the IV plus IP chemotherapy group (16.2% vs. 39.1%). One common cause of peritoneal dissemination is the metastasis of free tumor cells to the abdominal cavity through blood or lymph Thus, it is crucial to eliminate the free tumor cells in the peritoneum. IP administration is a selective, locally therapeutic method with special pharmacokinetic advantages including: i) allowing drugs to penetrate into the portal vein through absorption in the abdominal cavity in order to increase the drug concentration in the portal vein, leading to more efficient elimination of cancer cells in the portal vein and the liver parenchyma; ii) drugs are readily distributed to all sections of the abdominal cavity, facilitating the full access of drugs to suspended cancer cells; iii) drugs used in IP possess pharmacokinetic advantages such as high selectivity with localized application allowing constant and sustained high drug concentrations in the abdominal cavity, while limiting the access to circulation and reducing potential toxicity; and iv) drugs can simultaneously kill the growth factor-producing inflammatory cells and platelets in the abdominal cavity, thereby decreasing the growth of cancer cells. Increasing numbers of abdominal cancers are currently under study to determine the effectiveness of adjuvant IP chemotherapy^25^,^26^.

The present review found that more adverse events are associated with IV plus IP chemotherapy. The incidence of neutropenia, edema and neuropathy was significantly increased. However, the small sample size diminish the strength of this result.

Based on the GRADE system, critical outcomes: the quality of survival rate was “moderate”; important outcomes: the rate of metastases and adverse events were “low”. The level of evidence concerning survival rate was downgraded due to the risk of bias between included studies that may have been caused by inadequate randomization, allocation concealment in the studies. The level of evidence concerning the rate of metastases rate and adverse events was downgraded due to the risk of bias and inaccuracy (small sample size).

Currently, six systematic reviews^4^,^5^,^6^,^7^,^8^,^9^ were found that evaluated the treatment effect of IP chemotherapy for gastric cancer. All of these six reviews compared IP plus surgery with surgery alone with a similar purpose: to assess the effect of IP therapy for gastric cancer. However, our review is different in that we investigated whether IP chemotherapy was still necessary following systematic chemotherapy after surgery. Therefore, we included studies comparing IP plus IV chemotherapy after gastric cancer resection with IV chemotherapy alone after resection. Compared with the previous reviews, we only found five studies exploring this clinical issue, most of which were not included by the previous five systematic reviews. The search strategies in our review were developed by information specialists who used a comprehensive approach to searching for published studies. In addition, we used the GRADE system to assess the quality of evidence. Decision makers did not simply consider the benefits and harm associated with a particular intervention, but were primarily influenced by their level of confidence in the evidence. It has clearly been shown that ignoring the quality of evidence can result in inappropriate or even controversial recommendations^27^. We also applied an intention-to-treat analysis to deal with the missing data after randomization, and our conclusions were tested by a sensitivity analysis.

### Limitations

There are a number of limitations to this systematic review and meta-analysis that need to be acknowledged. Firstly, and perhaps most notably, only a small number of RCTs met the inclusion criteria, thus reducing the power of the analyses. Secondly, only studies in the English-language literature were included, so it is possible that relevant studies in other languages will be identified in the future. Third, though no statistical heterogeneity was detected in any of the trials included in the meta-analysis, generally, methodological and clinical heterogeneity is always present^28^. Finally, only patients in Asia were identified in the present study, which may result in local bias. Patients in other areas of the world should also be considered in future studies.

## Conclusion

There is insufficient high quality evidence in the current literature regarding the effect and safety of IV plus IP chemotherapy versus IV chemotherapy alone for the treatment of gastric cancer. Hence, the findings from this meta-analysis are by no means definitive. Nevertheless, the pooled results indicated that IV plus IP chemotherapy might be more effective in improving survival rate and the rate of metastases, although the incidence of adverse events was increased when compared with IV chemotherapy alone. Clearly, there is a need for high quality RCTs to establish the effectiveness and safety of IV plus IP chemotherapy for the treatment of gastric cancer.

## Additional Information

**How to cite this article**: Yang, S. *et al.* A Comparison of Intravenous plus Intraperitoneal Chemotherapy with Intravenous Chemotherapy Alone for the Treatment of Gastric Cancer: A Meta-Analysis. *Sci. Rep.* **5**, 12538; doi: 10.1038/srep12538 (2015).

## Figures and Tables

**Table 1:** Main characteristic of five included studies.

Author	Country	Diagnose	Participants		Treatment		Control	Reported outcomes
			Age (years)	T/C	IV/IV + Oral	IP		
Shimoyama 1999^13^ **	Japan	Diffused or intestinal gastric cancer Diffuse type/intestinal type: 46/41	mean ~56.1, sd~10.5	30/33	**IV:** cisplantin (80 mg) started from 2 weeks post operation	**Intraoperation:** MMC (10 mg/patient) +500 ml of normal saline perfused into the intraperitoneal cavity for 1 hour.	**IV:** cisplantin (80 mg) started from 2 weeks post operation.	Survival rate
					**Oral:** UFT(300 mg/day or 600 mg/d) for 2 years, started from 4 weeks post operation.		**Oral:** UFT (300 mg/day) for 2 years started from 4 weeks post operation	
Deng 2009^14^	China	Gastric cancer Stage Laurence classification: not reported.	28–74	44/41	**IV:** 5-FU 500 mg/m^2^·d + cisplantin 15 mg/m^2^·d + leucovorin 200 mg/m^2^·d, started from 2 weeks after surgery successive use of 5 days per month, for 4–5 months.	**Intraoperation:** 5-Fu 1000–1500 mg + mictomycin 20 mg + 3000 ml saline; 42 °C–43 °C, 1000–1500 ml for 60–90 min; 1000 ml fluid residue	**IV:** The same regimen with treatment group.	Survival rate Recurrence rate Metastases rate Adverse events.
						**Post operation:** 5-Fu 1000–1500 mg + 3000 ml saline, once/day for successive 4 days.		
Kang 2013^15^ *	Korea	gastric adenocarcinoma Diffuse type /intestinal type: 161/301	25–69	322/318	**IV:** MMC 15 mg/m^2^, started from 1 day after surgery; Cisplatin 60 mg/m^2^, started from 4 weeks after surgery for 6 months.	**Intraoperation** : Cisplatin 100 mg with 1L of normal saline intraperitoneally for 2 hours during surgery.	**IV:** MMn-C 20 mg/m^2^ started from 3–6 weeks after surgery.	Overall survival Relapse Free survival Adverse events.
		Mixed/unknown: 51/8			**Oral:** Doxifluridine 460–600 mg/m^2^/day, started from 4 weeks after surgery for 12 months.		**Oral:** the same regimen with treatment group	
Zhang 2007^16^	China	Advanced gastric cancer without metastases Laurence classification: not reported.	21–70	92/120	**IV:** 5- FU 10 ~ 15 mg/kg + MMC 0.1 ~ 0.15 mg/kg + adriamycin 0.5 ~ 1 mg/kg, started from 7–15days after surgery, once a week for 2 ~ 3 weeks, 4 weeks interval before next treatment course for 6 courses.	**Intraoperation:** MMC 30 mg + cisplatin 100 mg + 2000 ml distilled water, 43 °C–45 °C perfusion to abdominal cavity for 30 min	**IV:** the same regimen with treatment group.	Positive rate of peritoneal free cancer cell; Survival rate;
							**Intraoperation IP:** 2000 ml distilled water perfused to abdominal cavity for 30 minutes	
Zhao 2006^17^	China	Advanced gastric cancer	35–72	40/32	**IV: 2 months after surgery:** Routine Chemotherapy.	**Intraoperation IP:** 43 °C distilled water perfused to abdominal cavity for 15 min.	**IV:** Routine Chemotherapy, started from 1 months after surgery	Survival rate Relapse rate.
		Laurence classification: not reported.				**Post operation IP:** MMC-CH started from 1 week post operation	IP**: only intraoperation IP, no post operation IP.**	

Notes: I = Treatment group, C = Control group, IV = Intravenous chemotherapy, Oral = oral chemotherapy, IP = Intraperitoneal chemotherapy, MMC-CH = Mitomycin active carbon particle, UFT = tegaful and uracil, MMC = Mitomycin-C.

^*^We assumed missing binary data from this study.

^**^This study included 6 chemotherapy groups, of which 4 groups is eligible in this meta-analysis. We combined two control groups with the same treatment regimen as well as two treatment groups with only different dosage of oral UFT (300 mg or 600 mg).

**Table 2:** Grade-Summary of findings.

Quality assessment							No of patients		Effect		Quality	Importance
No of studies	Design	Risk of bias	Inconsistency	Indirectness	Imprecision	Other considerations	Surgery plus IP and IV	Surgery plus IV only	Relative (95% CI)	Absolute		
Survival rate-1-year survival rate												
3	randomised trials	serious^1^	no serious inconsistency	no serious indirectness	no serious imprecision	none	157/166 (94.6%)	168/194 (86.6%)	RR 1.1 (1.03 to 1.17)	87 more per 1000 (from 26 more to 147 more)	□□□□ MODERATE	CRITICAL
								84.9%		85 more per 1000 (from 25 more to 144 more)		
Survival rate-3-year survival rate												
5	randomised trials	serious^2^	no serious inconsistency	no serious indirectness	no serious imprecision	none	318/469 (67.8%)	271/484 (56%)	RR 1.22 (1.1 to 1.35)	123 more per 1000 (from 56 more to 196 more)	□□□□ MODERATE	CRITICAL
								56.7%		125 more per 1000 (from 57 more to 198 more)		
Survival rate-5-year survival rate												
3	randomised trials	serious^3^	no serious inconsistency	no serious indirectness	no serious imprecision	none	85/162 (52.5%)	69/185 (37.3%)	RR 1.42 (1.12 to 1.8)	157 more per 1000 (from 45 more to 298 more)	□□□□ MODERATE	CRITICAL
								37.5%		157 more per 1000 (from 45 more to 300 more)		
Metastases rate-Distant metastases												
1	randomised trials	no serious risk of bias	no serious inconsistency	no serious indirectness	very serious^4^	none	7/44 (15.9%)	16/41 (39%)	RR 0.41 (0.17 to 0.88)	230 fewer per 1000 (from 47 fewer to 324 fewer)	□□□□ LOW	IMPORTANT
								39%		230 fewer per 1000 (from 47 fewer to 324 fewer)		
Metastases rate-Peritoneal recurrence and metastases												
2	randomised trials	serious^5^	no serious inconsistency	no serious indirectness	no serious imprecision	none	22/136 (16.2%)	63/161 (39.1%)	RR 0.4 (0.25 to 0.63)	235 fewer per 1000 (from 145 fewer to 293 fewer)	□□□□ MODERATE	IMPORTANT
								40.7%		244 fewer per 1000 (from 151 fewer to 305 fewer)		
Adverse effect-Neutropenia												
2	randomised trials	serious^6^	no serious inconsistency	no serious indirectness	no serious imprecision	none	223/307 (72.6%)	165/299 (55.2%)	—	552 fewer per 1000 (from 552 fewer to 552 fewer)	□□□□ MODERATE	IMPORTANT
								36.1%		361 fewer per 1000 (from 361 fewer to 361 fewer)		
Adverse effect-Anemia												
1	randomised trials	serious^6^	no serious inconsistency	no serious indirectness	serious^7^	none	238/263 (90.5%)	230/258 (89.1%)	RR 1.02 (0.96 to 1.08)	18 more per 1000 (from 36 fewer to 71 more)	□□□□ LOW	IMPORTANT
								89.2%		18 more per 1000 (from 36 fewer to 71 more)		
Adverse effect-Vomiting/Nausea												
1	randomised trials	serious^6^	no serious inconsistency	no serious indirectness	serious^4^	none	10/44 (22.7%)	9/41 (22%)	RR 1.04 (0.44 to 2.05)	9 more per 1000 (from 123 fewer to 230 more)	□□□□ LOW	IMPORTANT
								22%		9 more per 1000 (from 123 fewer to 231 more)		
Adverse effect-Thrombocytopenia												
1	randomised trials	serious^6^	no serious inconsistency	no serious indirectness	serious^7^	none	62/263 (23.6%)	47/258 (18.2%)	RR 1.29 (0.92 to 1.81)	53 more per 1000 (from 15 fewer to 148 more)	□□□□ LOW	IMPORTANT
								18.2%		53 more per 1000 (from 15 fewer to 147 more)		
Adverse effect-Edema												
1	randomised trials	serious^6^	no serious inconsistency	no serious indirectness	no serious imprecision	none	74/263 (28.1%)	20/258 (7.8%)	RR 3.63 (2.41 to 5.14)	204 more per 1000 (from 109 more to 321 more)	□□□□ MODERATE	IMPORTANT
								7.8%		205 more per 1000 (from 110 more to 323 more)		
Adverse effect-Abnormal liver function												
1	randomised trials	no serious risk of bias	no serious inconsistency	no serious indirectness	very serious^4^,^8^	none	3/44 (6.8%)	2/41 (4.9%)	RR 1.4 (0.24 to 6.47)	20 more per 1000 (from 37 fewer to 267 more)	□□□□ LOW	IMPORTANT
								4.9%		20 more per 1000 (from 37 fewer to 268 more)		
Adverse effect-Neuropathy												
1	randomised trials	serious^6^	no serious inconsistency	no serious indirectness	no serious imprecision	none	165/263 (62.7%)	46/258 (17.8%)	RR 3.52 (2.97 to 4.02)	449 more per 1000 (from 351 more to 538 more)	□□□□ MODERATE	IMPORTANT
								17.8%		449 more per 1000 (from 351 more to 538 more)		
Peritoneal cancer free cell detection												
1	randomised trials	serious^9^	no serious inconsistency	no serious indirectness	no serious imprecision	none	6/92 (6.5%)	35/120 (29.2%)	—	292 fewer per 1000 (from 292 fewer to 292 fewer)	□□□□ MODERATE	IMPORTANT
								0%		—		

^1^one study assigned 17.4% weight has high risk in randomisation, although a potential risk of blinding,the detection or performance bias may be impossible to affect this outcome.

^2^80% results were derived from studies with high or potential risk of allocation concealment.

^3^all three studies have potential risk of allocation concealment or randomization.

^4^small sample size n = 85.

^5^The result is derived from two studies with potential risk in randomisation or allocation.

^6^High risk of allocation and blindness as the result derived from an open label study.

^7^small sample size n = 521 which is smaller than the optimal information size.

^8^wide confidence interval.

^9^Potiential risk of allocation concealment and blinding.

potential risk of blinding and allocation.
